# Cone Beam CT With Flat Panel Detector and Biplane Fluoroscopy-Guided Percutaneous Trigeminal Nerve Rhizotomy Using Three-Dimensional Needle Trajectory Planning

**DOI:** 10.7759/cureus.25538

**Published:** 2022-05-31

**Authors:** Dylan T Cohen, Ilya Bragin, Roy Hwang, Martin Oselkin

**Affiliations:** 1 Neurology, State University of New York (SUNY) Downstate Medical Center, Brooklyn, USA; 2 Neurology, Lewis Katz School of Medicine at Temple University, Philadelphia, USA; 3 Neurology, St. Luke's University Health Network, Bethlehem, USA; 4 Neurosurgery, St. Luke's University Health Network, Bethlehem, USA; 5 Neurosurgery/Interventional Neuroradiology, St. Luke's University Health Network, Bethlehem, USA

**Keywords:** gasserian ganglion, biplane fluoroscopy, percutaneous radiofrequency rhizotomy, glycerol rhizotomy, meckel's cave, neuronavigation, trigeminal neuralgia, trigeminal rhizotomy, needle trajectory planning, cone-beam computed tomography (cbct)

## Abstract

Trigeminal-mediated pain disorders can be devastating for patients refractory to medical therapy. Gasserian ganglion blocks and percutaneous trigeminal rhizotomy have been used with success to treat these patients, however, serious complication risks include facial hematoma, cranial nerve palsy, and stroke. Cone beam CT, combined with fluoroscopy and needle navigation has been shown to decrease needle pass rates, procedure time, radiation exposure, and complications in multiple interventional radiology procedures, but hitherto has not been utilized for Gasserian ganglion interventions. Here, we present two cases of trigeminal-mediated pain successfully treated via cone beam CT combined fluoroscopy and needle navigation.

## Introduction

Trigeminal neuralgia is a rare, recurrent, and debilitating pain disorder within the distribution of the branches of the trigeminal nerve. The pain is severe, unilateral, and shock-like. It is usually intermittent but some patients report continuous less intense baseline pain [1]. Painful trigeminal neuropathy can also present as trigeminal neuralgia-like syndrome. Carbamazepine is the initial treatment of choice in trigeminal neuralgia, however, additional medications may be employed [1]. For medically refractory trigeminal neuralgia cases, surgical options may be considered. Microvascular decompression via craniotomy has classically been employed, however, it is a complex and invasive therapy. In the past decade, percutaneous trigeminal rhizotomy (PTR) has emerged as an increasingly popular alternative to open surgery given its less-invasive nature [2]. 

PTR is an image-guided procedure that involves ablation of the nociceptive fibers of the fifth cranial nerve residing in the Gasserian ganglion. There are three commonly performed methods: balloon compression, glycerol injection, and radiofrequency ablation [2]. However, a temporary block may also be utilized when neurolysis is not desired. Techniques for each require an identical approach, whereas a needle is inserted lateral to the corner of the mouth and advanced toward the skull base and into Meckel’s cave via the foramen ovale. X-ray, CT, fluoroscopy, or some combination of each may be used to guide the needle into the proper position. Pain relief for all modalities of PTR ranges between 90% and 97% in the immediate post-operative time period and is durable with 54-66% of patients experiencing continued relief at three-year follow-ups [2]. Subtleties between the three may dictate operational choice on a case-dependent basis, however, the similarity in technique among each lends credence to the idea that exact needle placement with minimal needle manipulation and adjustments is critical for procedural effectiveness as well as for reduction of complications.

Classically, PTR is performed using anatomic landmarks and palpation to guide the needle into the foramen ovale. Intermittent fluoroscopy is used simultaneously to track needle advancement and confirm needle depth within Meckel’s cave [2]. Precise access to the foramen ovale is of utmost importance. Operators often utilize 18 gauge needles in these cases as it is easier to manipulate the needle into the foramen by walking the needle off the skull base. However, this is a technically challenging procedure and there are critical structures within millimeters of the foramen ovale such as the carotid artery, other cranial nerves, as well as the brain. Complications may include facial hematoma, carotid cavernous fistula, cranial nerve palsy, stroke, and intracranial bleeding [3,4]. New technologies have evolved that offer improved precision, efficiency, and safety in performing PTR such as CT, CT-fluoroscopy, and cone beam CT (CBCT). Cone beam CT, when combined with fluoroscopy and needle navigation, has been shown to decrease needle pass rates, procedure time, radiation exposure, and complications in multiple interventional radiology procedures [5]. While such a method has been utilized in practice for several other percutaneous cases (e.g., needle biopsies), the same cannot be said in application to trigeminal nerve blocks and rhizotomies [6,7]. Here, we present two patients suffering from trigeminally-mediated pain treated by a percutaneous approach utilizing fluoroscopy and needle navigation following CBCT. An IRB exemption at St. Luke's University Health Network is not required for case series under three cases.

## Technical report

Case 1

A 42-year-old female presented to the ED with a chief complaint of right-sided facial numbness, pain, and intractable headaches. Five months prior, she underwent a dental extraction for an impacted mandibular wisdom tooth. This was complicated by osteomyelitis for which she required multiple debridements and long-term IV antibiotics. She developed numbness four days following the last debridement, which was then followed by facial pain and severe right retro-orbital lancing pain and right frontal headaches. She was managed as an outpatient with a variety of medications including carbamazepine. An MRI with a trigeminal neuralgia protocol revealed a small vessel in contact with the inferolateral margin of the proximal cisternal right trigeminal nerve but no deflection or mass effect on the nerve. She had never experienced symptoms of trigeminal neuralgia prior to her mandibular osteomyelitis. Given this, and the significant, discrete sensory loss on examination, the patient was formally diagnosed with The International Classification of Headache Disorders, Third Edition (ICHD3) classification of painful trigeminal neuropathy attributed to other disorder, with a consideration that she may have had a “double crush” situation with some predisposition to pain given the mild chronic vascular nerve compression. Following two months of conservative therapy and dosage escalations, her symptoms progressed at which point she required hospitalization. A bedside transnasal sphenopalatine ganglion block was performed providing no relief. Ketamine infusion was also initiated but she was ketamine dependent and could not be titrated down without return of her pain. Given the trigeminal neuropathy, a percutaneous intervention was believed to be the best therapeutic option to help her pain. Given her baseline V3 sensory loss and that her headache pain was her most bothersome feature, it was decided that a Gasserian block rather than neurolysis would be best to ameliorate her pain and avoid potential anesthesia dolorosa. 

**Figure 1:**
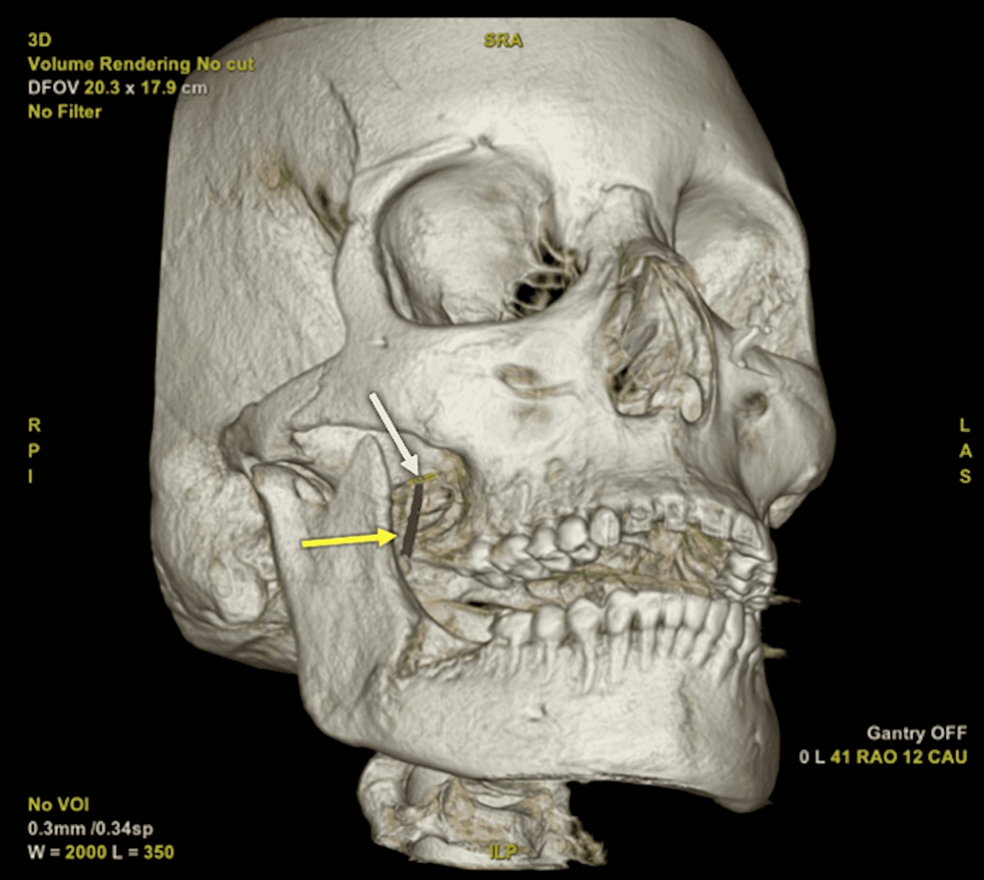
Three-dimensional volume rendered skull from cone beam CT with planned needle trajectory Foramen ovale (white arrow) identified as target for automated generation of needle guidance pathway (yellow arrow).

The procedure was performed under conscious sedation with monitored anesthesia care. The patient was placed supine on the angiographic table (IGS 630; Chicago, IL: GE Healthcare) with her head slightly extended and secured with paper tape. The AP detector was positioned in the ipsilateral oblique submental view to uncover the foramen ovale while the lateral detector was used to visualize the clivus. Next, using Hartel’s technique, a 3.5-in 22-gauge Quincke spinal needle entered the skin 2.5 cm lateral to oral commissure and was advanced towards the foramen ovale. The needle was advanced with counter-pressure from a finger in the oral cavity to ensure the mucosa was not violated. Once the needle was at the level of the posterior aspect of pterygoid plates, it was felt potential oral cavity entry was unlikely. Therefore at this time, a three-dimensional (3D) rotational cone beam CT was performed to aid in needle guidance. The needle tip was used as the entry point and foramen ovale was selected as the target point in order to create a trajectory line. Thereafter, the needle was advanced into Meckel’s cave using fused 3D-fluoroscopy imaging navigation (Needle ASSIST; Chicago, IL: GE Healthcare) (Figure 1).

**Figure 2:**
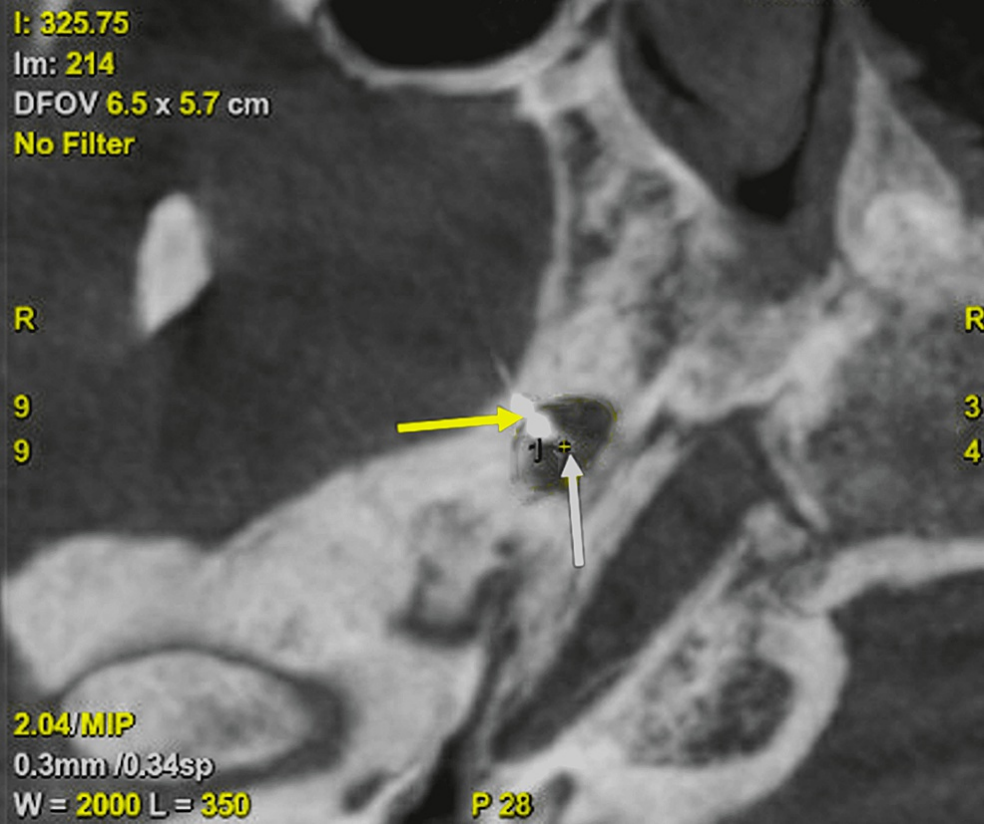
Cone beam CT skull for needle confirmation Insertion of needle tip identified (yellow arrow) through foramen ovale into Meckel’s cave (white arrow), confirming proper placement.

Three-dimensional rotational-cone beam CT was again performed and it confirmed needle tip passed through foramen ovale and terminated within Meckel's cave (Figure 2). The needle stylet was removed and it demonstrated a small amount of cerebrospinal fluid (CSF) egress. Contrast was injected under subtraction fluoroscopy to ensure no vascular uptake was present. Next 0.5 mL of a 50-50 mixture containing the 0.25% bupivacaine and 10 mg/mL dexamethasone was injected. The needle was removed. The patient tolerated the procedure well without complication.

The patient reported significant facial pain relief and complete resolution of headaches following the procedure and was discharged the next day. She continued to be headache-free two months post-procedurally. An ablative approach will be considered if the patient is still not experiencing long-term symptomatic relief.

Case 2

A 54-year-old male with a history significant for chronic alcoholism, hypertension, anxiety, and depression presented to the headache clinic with complaints of daily headaches for the past three months, associated with photophobia, phonophobia, and nausea. He described a constant pressure and sharp-shooting pain localized to his left head and face, more specifically in the frontal and temporal regions. At the time of initial presentation to his primary physician it was unclear if he was suffering from migraine without aura or an isolated trigeminal neuralgia, as predominant V1 involvement is atypical in trigeminal neuralgia and was therefore started on gabapentin. He initially found relief with gabapentin but this soon lost efficacy. Eventually he was formerly diagnosed by a neurologist with trigeminal neuralgia and carbamazepine as well as baclofen were added to his regimen. Ultimately he was requiring increasing doses of carbamazepine due to breakthrough pain and ultimately he developed severe hyponatremia. The severity of his discomfort had significantly reduced his quality of his life, decreasing his ability to sleep, his ability to open his mouth causing him to stop eating and lose weight, and causing him considerable mental affliction consistent with signs of depression. Medication failure and quality of life reduction were an indication at this time for a trigeminal nerve glycerol rhizotomy.

**Figure 3:**
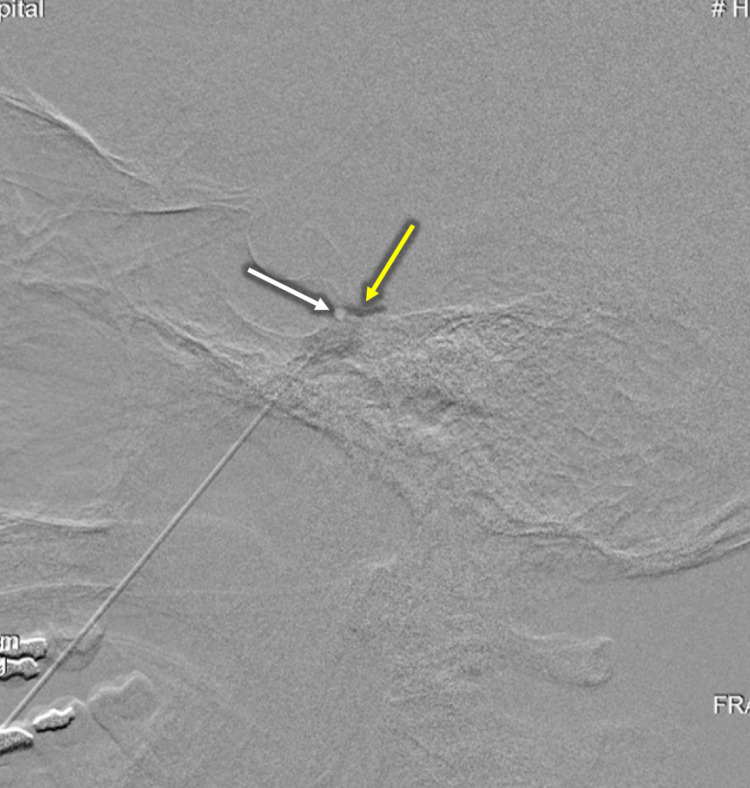
Digital subtraction CT for needle confirmation Contrast injection used to identify needle tip placement (white arrow) within opacified Meckel’s cave (yellow arrow).

The procedure was performed in an identical fashion to that described above in case 1 with the addition of a neurolytic agent. Once the needle position was confirmed by contrast injection under subtraction opacifying Meckel’s cave, the patient was transferred from the angiography table to a stretcher and was placed in the upright position with his head flexed. 0.2 mL of anhydrous glycerol was then injected and the needle was removed (Figure 3). The patient tolerated the procedure well without complication and was transferred to the post-anesthesia care unit (PACU) in stable condition. Due to the hyperbaric nature of glycerol to CSF, the patient remained upright for two hours with head flexed in order for the glycerol to coat all three divisions of the trigeminal nerve.

The patient reported complete resolution of facial pain and headaches following the procedure and was discharged the same day. He continued to be pain free three months post-procedurally. There was no new numbness or corneal anesthesia. His carbamazepine was eventually weaned off.

## Discussion

The goal of PTR is to accurately cannulate the foramen ovale such that the operator can subsequently treat the nociceptive fibers of the trigeminal ganglion. Ideally, the treatment should provide long-term pain relief as well, but the procedure should have minimal complications and side effects, of which there are several [8]. Improper cannulation or multiple attempts at correction have led to a variety of detriments, ranging from temporal and brainstem hematomas to blindness, carotid artery hemorrhages, and in worse case scenarios, death [9]. Although the noted side effects are unlikely a majority of the time, effective treatment for qualified patients is therefore contingent largely upon a refined technique. 

Modern practice of PTR usually relies on fluoroscopy as a means to gauge proper needle positioning. Although a trained eye can detect the foramen ovale on fluoroscopy, its shape and size differ on a patient-by-patient basis and clear views can be difficult to obtain despite a combination of anterior posterior oblique and lateral imaging. Often, the meticulousness of the procedure results in the operator needing to perform a number of trials until an adequate comfort level for preciseness can be generated, with confidence relying more on muscle memory than the aid of equipment [10].

Several reports have been documented that aim to improve operator confidence. Typically this means better procedural planning, improved image resolution, and confirmation of a properly executed technique. One report details the use of a single-plane, flat-panel detector system to accomplish this [10]. Another utilizes a biplane angiography system for a similar purpose [11]. Neuronavigation in conjunction with CT has also been implemented [12]. However, while each of these systems in isolation has its own benefits over conventional methodology, combining these strategies as we have done with the use of biplane fluoroscopy with CBCT and needle navigation may allow for optimal value to the patient, operator, and operating room staff if used routinely. 

Increased efficiency and accuracy in performing the procedures translates to fewer side effects. Optimization of technique lessens the requirement to maneuver the needle into prime positioning, decreasing the likelihood of inducing facial hematomas, a complication that occurs in 7% of cases. More serious complications such as carotid puncture or brain abscess, although rarer, could be considered even less likely with greater confirmation of needle placement [4]. Anesthesia toxicity is a major side effect that may occur, yet decreasing the time for the procedure will decrease this likelihood. Shorter procedural duration will by default mean less exposure to radiation, which is favorable for both the patient and operating room staff.

## Conclusions

Cone beam CT, when combined with fluoroscopy and needle navigation served as a novel, safe, and effective means of performing trigeminal nerve rhizotomy of the Gasserian ganglion. Success among a larger scale of patients should be replicated, however, for further demonstration of practicality and routine implementation.
